# Concurrent functional gastrointestinal disorders in patients with inflammatory bowel disease

**DOI:** 10.3389/fgstr.2022.959082

**Published:** 2022-08-12

**Authors:** Caroline Walker, Anna Boland, Andrew Carroll, Anthony O’Connor

**Affiliations:** Gastroenterology Department, Tallaght University Hospital, Dublin, Ireland

**Keywords:** inflammatory bowel disease, functional gastrointestinal disorder (FGID), irritable bowel syndrome, gut brain axis, gastrointestinal disease

## Abstract

Approximately 25% of people with quiescent inflammatory bowel disease (IBD) have symptoms caused by a functional gastrointestinal disorder (FGID). The pathophysiology of FGIDs in IBD is multifactorial. The gut–brain axis plays an important role as a bidirectional pathway with reciprocal gastrointestinal and psychological symptoms. Other factors include altered gastrointestinal motility, microbiome dysbiosis, medication use, prior surgery, impaired intestinal permeability, immune-system activation, and visceral hypersensitivity. As both IBD and certain FGIDs can have similar symptoms, it can be difficult to determine which disorder is the precipitant of symptoms. However, a prompt diagnosis of an overlapping FGID helps avoid unnecessary corticosteroid use and escalations of IBD treatment. Despite their prevalence, there have been very few randomized controlled trials conducted on therapeutic interventions for overlapping FGIDs in IBD. Therefore, management usually follows those interventions recommended for FGIDs, with certain adaptations made to allow for an altered gastrointestinal anatomy and functioning, caused by IBD.

## Introduction

Functional gastrointestinal disorders (FGIDs) are disorders of chronic and recurring gastrointestinal symptoms, which occur due to abnormal functioning of the gastrointestinal tract. They are considered disorders of gut–brain axis dysregulation and are associated with pathophysiologic factors such as dysbiosis, dysmotility, visceral hypersensitivity, and altered central nervous system processing (1). FGIDs account for approximately 40% of gastrointestinal problems seen by doctors and therapists (2). They are the most common gastrointestinal disorders in the general population; a 12-year longitudinal study on the prevalence of FGIDs reported an incidence of 42% (1).

The diagnosis of FGIDs is made by the application of the Rome IV criteria to symptoms present. The diagnosis should be a positive one, rather than a diagnosis of exclusion. As FGIDs occur in the absence of pathognomonic radiological, biochemical, or histopathological findings, investigations should be determined by symptoms or patient factors that predispose or suggest organic gastrointestinal disease. The presence of anxiety and depression is a risk factor for the development of an FGID, and if present, patients benefit from their treatment (3, 4). However, the mainstay of treatment is the management of the predominant symptoms.

Inflammatory bowel disease (IBD) is a chronic inflammatory condition of the gastrointestinal tract. It comprises two disorders: Crohn’s disease (CD) and ulcerative colitis (UC). There can be clinical similarities between the presentations of IBD and FGIDs. Both IBD and FGID have similar bowel symptoms that occur with variable severity, and both conditions follow chronic relapsing–remitting courses. Thus, in people with both IBD and an FGID, it can be difficult to determine which disorder is the cause of a symptom, particularly as there can be a poor correlation between mucosal inflammation and symptoms experienced in IBD (5). It is also important to note that symptoms caused by FGID have a negative impact on wellbeing and quality of life similar to that of symptoms caused by active IBD (6).

Approximately 25% of IBD patients with quiescent disease have gastrointestinal symptoms caused by a FGID (7). The pathophysiology of FGIDs in IBD is multifactorial, with post inflammatory changes occur in IBD likely contributing to their development. A number of these factors may occur and combine to cause abnormal functioning of the gastrointestinal tract. These include altered gastrointestinal motility, microbiome dysbiosis, medication use, prior surgery, impaired intestinal permeability, immune-system activation, and visceral hypersensitivity (8).

The shift in IBD management to a “treat to target” approach has heightened awareness among gastroenterologists of the need to correlate symptomatology with evidence of active ongoing disease. Active inflammation can now be identified with newer less invasive techniques such as stool calprotectin testing, capsule endoscopy, and imaging. These techniques can contribute to a prompter diagnosis of an overlapping FGID as the precipitant of a symptom, which helps avoid unnecessary and potentially harmful corticosteroid use and escalations of IBD treatment.

## Specific problems

The Rome IV criteria divide FGIDs into six subcategories based on their anatomical origin: esophageal disorders, gastroduodenal disorders, bowel disorders, centrally mediated disorders of gastrointestinal pain, gallbladder and sphincter of Oddi disorders, and anorectal disorders (9). FGIDs are then further divided into symptom-based groups. This classification enables a standardized approach for diagnosis and management.

In studies with a direct comparison, FGIDs appear to be more prevalent in people with quiescent IBD compared to the general population (3). Data on the prevalence of FGIDs in IBD is variable. A 2020 meta-analysis of people with IBD experiencing IBS symptoms showed that the prevalence of IBS symptoms is lower when IBD remission is determined by endoscopy or histology findings (23.5%), compared to validated disease activity scores (33.6%) (10). In people with quiescent IBD but refractory defecation symptoms, anorectal FGIDs are an important differential to consider, as they are commonly the cause of these persisting symptoms (11). IBS is the most commonly occurring FGID with CD and appears to be significantly more prevalent in CD compared to UC. However, this possible exaggeration could be partly explained by the symptom profile of CD being the most similar to IBS, combined with the relatively less available investigations that can objectively rule out subclinical inflammation in the small bowel (3, 7). Functional constipation is the most frequently occurring FGID in UC (3). Other FGIDs seem to occur equally in both forms of IBD.

When compared to those with IBD alone, people with IBD and an overlapping FGID have higher rates of anxiety, depression, and somatization (3, 12). They also have a poorer quality of life (4). This is multifactorial, with research suggesting that people with concurrent IBD and FGIDs may have more restricted leisure times and sleep disturbances, and may be more fatigued. In addition, they may have less social satisfaction, be less productive at work, and have higher rates of absenteeism (3, 12, 13). They also attend general practitioners and gastroenterology services more frequently, undergo more medical investigation, have higher rates of opioid use, and have higher healthcare costs than those with IBD alone (3, 12). In spite of this, a longitudinal study that followed people with concurrent IBS and IBD symptoms over a 2-year period found that, at 2 years, these people had similar incidences of inflammatory complications such as flares, treatment escalation, hospitalization, and surgery, when compared to their counterparts with IBD alone. This indicates that the increased costs of care, inferior quality of life scores, and other negative features of concurrent FGID with IBD do not appear to have an inflammatory basis (3, 14). Much research therefore has focused on whether or not psychological factors may be the key driver of many of the problems described.

## Gut–brain axis

There are higher rates of depression and anxiety with IBD and FGIDs (15). Not only does IBD predispose to these disorders, but psychological stress itself can precipitate flares in IBD through stimulation of the gut–brain axis (16). Approximately 50% of patients with comorbid psychological conditions and FGIDs develop gastrointestinal symptoms before they develop the psychological symptoms that lead to the diagnosis of a psychological disorder (17). This supports the theory of the gut–brain axis as a bidirectional pathway with reciprocal gastrointestinal and psychological symptoms, and implies that FGIDs may predispose people to anxiety and depression, and psychological conditions may predispose people to FGIDs.

Animal models of the gut–brain axis in depressed and stressed states have found increased intestinal permeability, and increased clinical and subclinical immune activation (18). This is largely mediated through the HPA axis, with corticotropin-releasing hormones amplifying and modulating the immune response (19). Another contributing factor is visceral hypersensitivity, which occurs due to altered central processing of stimuli, and is supported by the finding that people with premorbid anxiety have higher rates of post-infectious IBS (20). Psychological stress may also alter the gut microbiome, with early life stress in rats being shown to be associated with altered microbiota, visceral hyperalgesia, increased inflammatory responses, and higher levels of adrenocorticotrophin (ACTH) (21).

In the other direction, dysbiosis can have an effect on the brain through a variety of methods. One example is the increase in ACTH and other stress hormones seen in germ-free mice, that decrease with the manipulation of microbiota, suggesting that a diverse and robust gut microbiota is protective against high levels of ACTH (21). Additionally, multiple enteric neurotransmitters are produced, in part, by the microbiome and therefore dysbiosis can drive disordered levels of ƴ–aminobutyric acid (GABA), serotonin, glutamate, histamine, and other neurotransmitters (22). This partially explains altered visceral sensation and gut motility from a locally acting neurotransmitter. Circulating inflammatory cytokines also play a central role, as they are increased in both IBD and in functional gastrointestinal diseases. Studies have shown that even in quiescent IBD, anti-tumor necrosis factor α (anti-TNF-α) treatment can improve visceral sensitivity and symptoms severity through central processes, with alterations in the limbic system on functional MRI (23). Overall, there is little doubt that while the brain can alter gut homeostasis to drive gastrointestinal symptoms, alterations in gut physiology can influence changes in brain functioning. Therefore, the psychological comorbidity that we see in this cohort of patients not only significantly influences quality of life, but also is a key component of the disease process, and needs to be considered when assessing patients.

## Pathogenesis

The current understanding regarding the pathogenesis of IBD largely centers around the interplay of genetic factors, an altered gut microbiota triggering an abnormal host immune response, environmental factors such as smoking and diet, and immunological dysregulation (24). Similar concepts can be seen in the understood pathophysiology of FGIDs. It raises the question as to whether the strong correlation in our patients with IBS and FGIDs is a result of direct causation alone, or in fact down to shared predisposing factors.

The mechanisms of FGIDs are incompletely understood and vary depending on the subtype. The pathogenesis of IBS is the most well described and many of the core concepts are echoed in pathogenesis of all other FGIDs. Symptoms are thought to be driven by visceral hypersensitivity and gastrointestinal motor disturbance. The underlying drivers and contributors to these phenomena are varied, with an interplay of genetic factors, disruptions in the gut microbiota, disordered immunogenicity and low-grade inflammation in the gut, food sensitivities, and central dysregulation being implicated. The heterogeneity of the phenotypes of FGIDs speaks to the likelihood that there are many different underlying causes of these disorders. This also raises the potential for therapies to be developed and chosen based on the underlying mechanism of the disorder rather than the symptom complex involved (23).

Genetic risks for IBS have only recently been identified. A large study published in 2021 showed a significantly higher incidence of IBS in monozygotic twins compared to dizygotic twins, implying a genetic component to IBS. Interestingly though, that same study identified that having a mother or father with IBS was actually a stronger risk factor for having IBS than having a twin with the condition, implying a stronger role for social learning in the development of the condition (25). Specific genes have also been implicated; for instance, missense mutations in SCN5A, which alter the function of voltage-gated mechanosensitive Na- channels in the gut, are present in up to 2% of IBS sufferers, and this has been used as a target to tailor treatment (26). Similarly, variations on chromosome 9 (also the chromosome in which variants are implicated in IBD), particularly variants at the locus 9q31.2, have been associated with an increased risk of IBS in women (27). Heritability has also been loosely implied in functional dyspepsia, although this seems to be only a low-level risk factor (5% heritability) and it seems the predisposition to functional dyspepsia is shared with a predisposition to multiple other conditions across systems (28).

The specific pathogenesis of overlapping FGIDs with IBD has not yet been fully elucidated. Within the context of overlapping IBS and IBD, several potential mechanisms may be identified. Many of these mechanisms occur in active IBD and persist with remission, possibly due to the effect of chronic inflammation. These include functional changes in motility and absorption, abnormalities of the enteric nervous system affecting motility, and increased intestinal permeability. Increased intestinal permeability impairs the functioning of the gut wall barrier, allowing translocation of luminal contents and consequently triggers an inflammatory response (29). Interestingly, a 2017 study by Chang et al. found that in people with IBD in remission, impaired intestinal permeability was associated with ongoing bowel symptoms, and increased permeability correlated with an increase in the severity of diarrhea experienced (30).

Microbiome dysbiosis is another possible element in the pathogenesis of FGIDs and IBD. IBD patients have been found to have a significantly reduced diversity of microbiota and dysbiosis of the microbiome (31). Similar microbiome patterns have been found in patients with FGIDs (32). Studies suggest that a higher concentration of *Clostridium* and *Bacteroides* species in particular, as found in these conditions, triggers an altered immune response with higher levels of T cells in gut mucosa (33). Research specifically examining alterations in the microbiome in people with quiescent IBD and IBS-type symptoms is sparse. A 2018 study by Stutkever et al. found that IBS-type symptoms were not associated with any significant distinct alterations of the microbiome composition at a phylum level (34). A later study by Cui et al. in 2021 had a similar finding at a phylum level for CD and UC, and at genus level for UC. However, when comparing the microbiome composition at the genus level in participants with quiescent CD, they found that those with IBS-type symptoms had an increased abundance of *Faecalibacterium* and a decreased abundance of *Fusobacterium*, when compared to those without IBS-type symptoms (35). Overall, in this developing area of research, it is too early to comment upon a causal relationship between alterations in the microbiome and the development of IBS with pre-existing IBD. However, further research examining alterations in microbiome compositions and therapies that modulate the microbiome, such as probiotics, in people with concurrent IBS and IBD, could hopefully contribute to improved understanding of the pathogenesis, and reveal future therapeutic options.

Finally, diet may also contribute to symptom development in FGIDs. Fermentable oligo-, mono-, and disaccharides and polyols (FODMAPs) have been associated with osmotic and fermentation effects, and consequently cause small bowel distension due to increased bowel water content (36). This can cause symptoms in those who suffer from the visceral hypersensitivity that occurs in FGIDs. Food intolerances, particularly gluten, have also been implicated as possible symptom precipitants in FGIDS, although studies have shown inconsistencies in intolerances once the foods involved have been reintroduced in a double-blinded manner. Lindi et al. conducted a large questionnaire based study to examine people with IBD perceptions about the effect of their diet on symptoms. They reported that 48% of participants believed that diet was the triggering factor for the development of IBD, one third of believed diet to be more important in influencing the disease course than medication, and 56% restricted their diet to avoid triggering foods or drinks (37). This concept of triggering foods or diets is not supported by our current scientific understanding of the disease. People with IBD often respond to their diagnoses by excluding sugars and increasing fiber intake, which has been shown to have no effect on the clinical course of IBD, but in some cases may worsen IBD symptoms or cause difficulty with CD strictures (38, 39). These dietary strategies are so emphasized potentially because of the influence they have on overlapping functional disorders rather than the impact on the disease itself.

## Diagnosis

In the absence of active inflammation and organic pathologies that mimic FGIDs, FGIDs can be diagnosed clinically by the Rome IV criteria. The exclusion of mucosal inflammation has become a hallmark of how we assess and quantify the problem of FGID symptoms in people with IBD (40). It is, however, difficult to conceive that problematic FGID symptoms, which are so common throughout all population groups, could not occur concurrently with active IBD. It is likely therefore that this distinction is purely arbitrary and more to do with the expediency for the academic community rather than addressing the real issues facing people and practitioners (29).

The first step in diagnosis is a detailed clinical assessment. The characteristics, duration, and severity of the concerning symptoms should be fully elucidated. The patient’s previous disease activity and the acuity of new symptom onset should be considered. Then, the presence of any other symptoms or recent risk factors that could suggest either an IBD flare or alternative organic pathology should be asked about. These include symptoms, such as bleeding, pain, high-frequency diarrhea, weight loss, fever, nocturnal symptoms, incontinence, or palpable masses, and risk factors like recent antibiotic use or sick contacts. Presenting symptom characteristics that align with the diagnostic criteria of the corresponding FGID should also be considered (**Table 1**). Additionally, any patient risk factors for the development of a condition to which IBD predisposes and has similar symptoms, such as bile acid diarrhea (BAD), small intestinal bacterial overgrowth (SIBO), or exocrine pancreatic insufficiency, should also be taken into account (41).

**Table 1:** A comparison of symptoms in IBS and active IBD.

Symptoms that can occur in both IBS and active IBD	Symptoms more suggestive of IBS	Symptoms more suggestive of active IBD
Abdominal pain	Bloating and gas	Blood in stool
Cramping	Constipation	Nocturnal diarrhea
Diarrhea	Variable stool consistency	Incontinence
Mucus in stool		Weight loss
Urgency		Arthralgia
Tenesmus		Pyrexia
Fatigue		Palpable masses

A physical examination should be performed. This should include a general examination for clinical signs suggestive of extra-intestinal manifestations of IBD; an abdominal examination to check for tenderness, palpable masses, distention, and bowel sounds; a rectal exam for perianal disease; and, if anorectal symptoms are not explained by this examination, a digital rectal examination for a rectal mass or defecation disorder.

The next step is to assess for active inflammation. Non-invasive markers should be used first. C-reactive protein (CRP) is a serum acute phase reactant. It can be elevated in the presence of active mucosal inflammation. However, its levels rise in response to a myriad of infectious and inflammatory conditions, so it is not a specific marker. Additionally, up to 15% of those with active IBD will not have a corresponding rise in CRP, and when it is elevated, the degree of elevation does not correlate accurately with mucosal inflammation (29, 42). Thus, CRP alone is not sufficient to rule out active inflammation, but it can be a helpful adjunct investigation. Calprotectin, a neutrophil protein, can be used as a surrogate marker of intestinal inflammation and is detectable in stool. However, fecal calprotectin can also rise in response to any condition that causes gastrointestinal intestinal inflammation including infection, ischemia, malignancy, or medications such as nonsteroidal anti-inflammatory drugs (NSAIDs). It may also be elevated in obesity. Overall, it is more sensitive (85%) and specific (75%) than CRP in assessing IBD activity, particularly in UC compared to CD (43, 44). It provides a more accurate reflection of inflammation by correlating well with the presence or absence of inflammation, as well as degree of disease activity (45). However, there is currently no clear consensus on what level of calprotectin is associated with remission in IBD. One large Canadian study suggests that FC ≥170 µg/g predicts endoscopic activity and FC ≥135 µg/g predicts histological activity in UC (46), whereas in CD, where more confounders exist, one study illustrated that a value under 225 mcg/g should be considered predictive of histologic remission (47). Overall, if the symptoms are more suggestive of active IBD or if calprotectin levels are borderline, then endoscopy with biopsies be performed. In people with CD who have a normal ileocolonoscopy, MR enterography or small bowel capsule endoscopy could also be performed to investigate for small bowel active inflammation or fibrostenotic disease.

Once the absence of active inflammation has been objectively proven, other IBD-associated aetiologies should be considered based on the presenting symptoms. BAD presents with watery postprandial diarrhea, urgency, incontinence, and bloating. Over one-third of people diagnosed with IBS with predominant diarrhea have undiagnosed BAD (48). It usually occurs due to bile acid malabsorption, which may occur in ileal CD and is almost universal with ileal resections (49). It can also be idiopathic or secondary to other malabsorptive gastrointestinal disorders such as cholecystectomy, coeliac disease, post-radiation, chronic pancreatitis, or SIBO (50). The availability of diagnostic testing is variable. Currently, the most common methods of diagnosis are a therapeutic trial of a bile acid sequestrant or a ^75^SeHCAT nuclear medicine scan.

The symptoms of SIBO are non-specific and similar to IBS and active IBD, often presenting with abdominal cramps, diarrhea, and bloating. It is slightly more common in CD compared to UC, and is concurrently present in almost one-third of CD people (29). Particular risk factors for development of SIBO in CD include fibrostenotic disease and multiple gastrointestinal surgeries (51). It can be diagnosed following a positive hydrogen or methane breath test or following a successful therapeutic trial of antibiotics. Another condition to consider is exocrine pancreatic insufficiency, which is more common in UC than CD, affecting approximately 22% and 14%, respectively. It presents with abdominal pain and steatorrhea, and the first investigation performed is a fecal elastase. Finally, other malabsorptive disorders including carbohydrate, lactose, and fructose malabsorption may also be more common in IBD and could be considered in the setting of suggestive symptom patterns (29).

FGIDs can be diagnosed if they fulfill Rome IV criteria. For IBS to be diagnosed, the presence of at least two of the following is needed: abdominal pain related to defecation, change in stool frequency, and change in stool appearance (4). Symptom onset must be at least 6 months prior to diagnosis, and these symptoms must be present for at least 1 day a week for the last 3 months (52). Based on the presenting symptoms, IBS can be further classified into subtypes: IBS with predominant diarrhea, IBS with predominant constipation, IBS with mixed bowel habits, and IBS unclassified.

Functional constipation can be considered if the symptoms do not meet the criteria for IBS with predominant constipation or opioid-induced constipation. For diagnosis, at least two of the seven criteria are needed. They must be present for the last 3 months with symptom onset more than 6 months prior to diagnosis. These criteria include the presence of straining, lumpy or hard stool, the sensation of incomplete evacuation, the sensation of anorectal blockage, or the need for manual maneuvers for more than 25% of defecations. Other criteria are having less than three spontaneous bowel motions a week or rarely having loose stools without the aid of laxatives (52). Of note, another differential for people with constipation, and UC in particular, is proximal constipation. Proximal constipation most commonly occurs with active left-sided disease, as the active distal disease contributes to fecal stasis in the proximal bowel. This results in constipation and constipation-associated symptoms, along with symptoms secondary to active disease (53). Rome III described the criteria for this disorder, which requires the presence of two or more symptoms present for more than 3 days during at least 3 months. These symptoms include bloating, excessive or troublesome flatus, abdominal cramps, decreased frequency of defecation, hard stool, straining with defecation, and sensation of incomplete defecation (52, 53).

Functional defecation disorders are a common cause of persistent defecatory symptoms in quiescent IBD (11). Diagnosing an overlapping functional defecation disorder with IBD can be difficult, as they can present with similar symptoms to those that occur with active IBD or with concurrent diarrhea, urgency, and incontinence. Thus, identifying the characteristic constipation and specific defecatory symptoms can be difficult (8). According to the Rome IV criteria, to diagnose a functional defecation disorder, a patient’s symptoms must first meet the criteria to be diagnosed with either IBS with predominant constipation or functional constipation. Symptom onset must be more than 6 months prior to diagnosis and symptoms must be present for the last 3 months (52). Additionally, there must also be objective evidence of impaired evacuation with an abnormal balloon expulsion test, anorectal manometry, or defecography (8). Two further functional defecation disorder subtypes are dyssynergic defecation and inadequate defecatory propulsion. Dyssynergic defecation is the most common of these functional defecation disorders (54). It can be diagnosed by the presence of inappropriate contraction of the pelvic floor muscles on anorectal manometry or anal surface electromyography (52). Inadequate defecatory propulsion can be diagnosed if there is evidence of inadequate propulsion on anorectal manometry.

**Figure 1:**
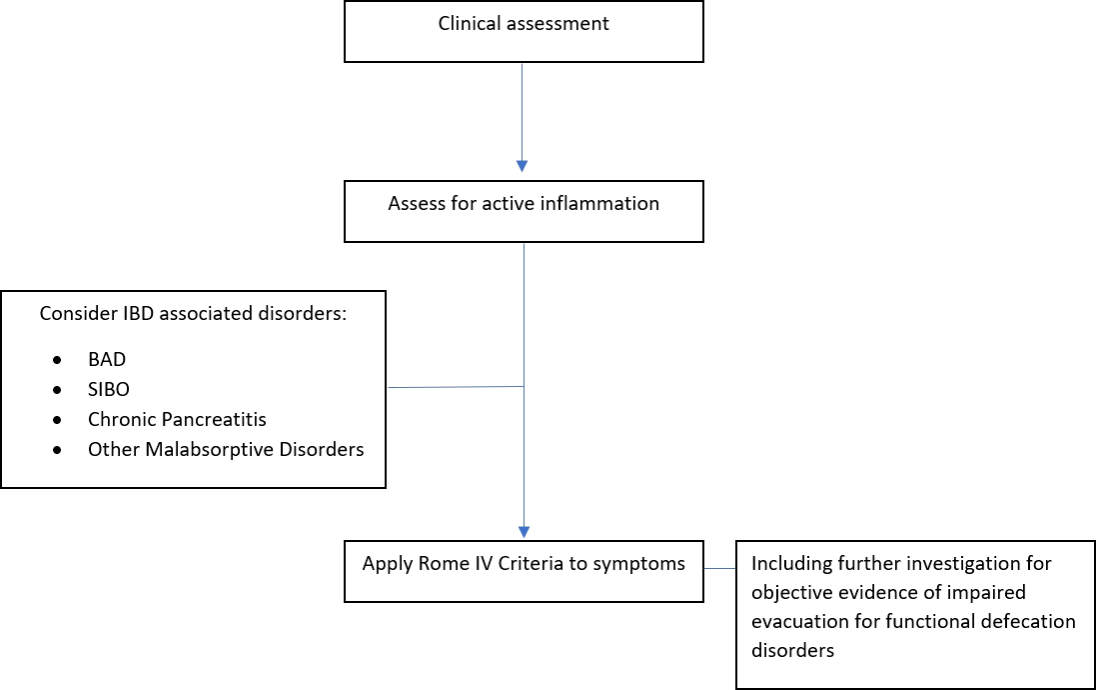
Suggested diagnostic algorithm for FGIDs in IBD.

In summary, once active inflammation has been excluded and other organic disorders with similar symptoms have been considered, a FGID can be diagnosed if the presenting symptoms fulfill the Rome IV Criteria (**Figure 1**). At diagnosis, it is important to provide the patient with a positive diagnosis, explained in a clear and understandable manner.

## Management

FGIDs are best managed with a combination of dietary, lifestyle, psychological, and pharmacological interventions, which are tailored to the patient. Unfortunately, there have been very few randomized controlled trials conducted on therapeutic interventions for overlapping FGIDs in IBD, and IBD patients are generally specifically excluded from interventional studies in IBS. Therefore, with concurrent IBD, empirical FGID management may need to be adjusted and tailored to the patient and their IBD (52).

FGIDs often follow a relapsing–remitting course and treatment approaches require significant patient engagement. Thus, it is essential to establish a good patient–doctor relationship. Open communication with validation of symptoms and shared discussion about treatment options is vital to ensure engagement with treatment.

The first step on the treatment pathway of FGIDs is dietary and lifestyle modifications. For people with bowel FGIDs, the first-line dietary changes that can be encouraged are eating regular meals, maintaining good hydration, reducing alcohol and caffeine intake, and limiting processed food (55). Subsequent advice can be tailored to symptoms. Patients with diarrhea should limit intake of foods containing fructose, sorbitol, mannitol, or xylitol, and reduce fiber intake. If constipation is the predominant symptom, increased dietary fiber intake with adequate hydration should be encouraged. Where bloating is causing discomfort, patients should decrease intake of beans and pulses if present, and could consider incorporating linseeds into their diet (55).

A second-line dietary intervention that could be considered in concurrent IBS with IBD is a dietician-led low-FODMAP diet. This is a second-line IBS treatment that has been shown to improve IBS symptoms (55). Current research on the effect of the FODMAP diet in people with IBS–IBD overlap is sparse. However, it may provide symptomatic benefit in people with persistent IBS symptoms with quiescent CD (29, 56). A small Danish randomized controlled trial conducted by Pedersen et al. with 78 participants, 37 of whom adhered to the FODMAP diet for 6 weeks following nutritionist advice and education, found that the participants with quiescent CD had an improvement in their IBS symptoms (measured by IBS-SSS). No improvement was seen in patients with UC or in those with mild–moderate IBD disease activity (7). Overall, the use of this restrictive diet in IBD patients should only ever be trialed cautiously and with the guidance of a dietician, as IBD patients are already at risk of malabsorption, poor nutrition, and nutritional deficiencies (52).

Exercise should also be encouraged as it has been shown to improve IBS symptoms, particularly constipation (57). It also may improve fatigue, depression, anxiety, and quality of life in IBS patients (58). In IBD, recreational exercise has been shown to reduce fatigue and relapse rates (59, 60). Although there are no studies that have specifically investigated the effects of exercise on overlapping FGIDs with IBD, it is likely that exercise in the presence of both disorders has a beneficial effect.

If symptoms persist despite dietary and lifestyle changes, then pharmacological treatment for individual symptoms can be considered for bowel FGIDs. As conventional analgesia is rarely effective for the abdominal cramps caused by gut wall dysmotility and spasm that occur in IBS, the first-line pain-relieving treatments recommended are peppermint oil and antispasmodics such as hyoscine butylbromide. Peppermint oil is well-tolerated, with less side effects compared to anti-spasmodics. However, studies show that both treatments improve abdominal pain, and contribute to an improved quality of life in IBS (55). For constipation, laxatives are recommended. Both stimulant and osmotic laxatives have been shown to be efficacious treatment options in patients with chronic constipation (61). While lactulose should be avoided due to bloating, no other particular laxative is recommended, so the choice should be determined by patient factors (55).

Despite a paucity of evidence to support its efficacy in IBS, loperamide is the first-line antidiarrheal medication recommended (55). However, the use of this medication in IBD needs to be very cautious as it can increase the risk of toxic megacolon, particularly in the setting of active IBD. In IBD patients with quiescent disease and chronic diarrhea or high-output stomas, loperamide can help reduce loss of fluid and electrolytes, and improve symptoms (62). Overall, in those with concurrent IBD and IBS predominant diarrhea (IBS-D), where active disease has been ruled out as the cause of the diarrhea, where neither BAD nor SIBO is felt to contribute to symptoms, and where IBS-D is thought to be the precipitant of diarrheal symptoms, loperamide could be considered as a treatment option, if dietary and lifestyle modifications have failed to improve symptoms.

If symptoms continue to persist, second-line pharmacological treatments can be considered. For global IBS symptoms, tricyclic antidepressants (TCAs) and selective serotonin reuptake inhibitors (SSRIs) are recommended due to their effect on the gut–brain axis. British National Institute for Clinical Excellence (NICE) guidelines recommend TCAs as the preferred option, commenced at a low dose, with up-titration if inadequate symptomatic response is noted (63). If symptoms fail to respond, then the TCA should be stopped and an SSRI can be trialed (55). Antidepressants have been shown to reduce symptoms in those with IBS (64). However, there is mixed evidence to support the use of antidepressants in IBD. Research by Hall et al. has suggested that in those with IBD who have abnormal anxiety and depression scores, those on antidepressants have lower scores in some markers of disease activity. Similarly, a small randomized controlled trial of 35 participants with quiescent IBD conducted by Daghaghzadeh et al. showed that a 12-week trial of a serotonin-norepinephrine reuptake inhibitor (SNRI) was associated with lower symptom severity scores and better quality of life scores (65). Conversely, a smaller randomized controlled trial conducted by Mikocka-Walus et al., on the effects of an SSRI on CD, found no effect on disease activity or quality of life (66). Although it is not clear whether antidepressants can be beneficial in IBD alone, they may be beneficial in the presence of anxiety or depression with IBD, and have been shown to reduce symptoms in IBS. It is therefore reasonable to suggest that in those with concurrent IBD and IBS, TCAs or SSRIs could be considered as a second-line pharmacological therapy.

The availability of second-line pharmacological therapies for IBS with predominant diarrhea is variable. These treatments include the antibiotic rifaximin, a mixed opioid receptor agent eluxadoline, and 5HT_3_receptor antagonists such as ramosetron (55). Finally, for patients with FGIDs causing constipation, secretagogues can be considered where symptoms persist despite the use of osmotic and stimulant laxatives. Secretagogues increase gastrointestinal transit and soften stool by increasing gut wall fluid and electrolyte content (55). Studies have shown that in addition to treating constipation, they also relieve abdominal pain and improve global IBS symptoms (55, 67).

An adjunctive treatment option for overlapping FGID in IBD that should be considered is psychological therapy. Multiple studies have shown that psychological therapy is an efficacious treatment in IBS (68, 69). The therapies with the largest evidence bases and suggested benefits over a longer term are cognitive behavioral therapy (CBT) and gut-directed hypnotherapy (55, 67). In IBD, CBT can have a positive effect on chronic pain and quality of life (70). In fact, the 2019 British Society of Gastroenterology consensus guidelines for IBD management recommend that psychological therapies should be offered as an adjunctive treatment to interested patients, particularly those with psychological symptoms (70). However, the optimal time to implement psychological therapies is unclear. In IBS, most guidelines suggest its use be reserved for IBS refractory to medical therapies after 12 months, while other studies support its early implementation as part of an interdisciplinary treatment approach (63, 71, 72).

Probiotics are another adjunctive treatment that can be considered for patients with IBS. Probiotics alter the microbiome and have been shown to improve abdominal pain and global symptoms in IBS (55). These improvements are usually small (29). Although the most efficacious combination of bacterial strains remains unclear, combination probiotics are more effective than single-strain probiotics. The use of probiotics in patients with concurrent FGIDs and IBD has not been specifically studied, and thus, the efficacy of their use in this cohort remains unclear.

A therapy to consider in patients with anorectal FGIDs is biofeedback therapy. Biofeedback therapy is a neuromuscular training therapy in which patients develop an improved perception of anorectal sensation and better muscle coordination for defecation with synchronized anal sphincter relaxation (8). It is a well-tolerated treatment that has been shown to improve symptoms and quality of life in patients with functional anorectal disorders. It is particularly effective with fecal incontinence and function defecation disorders (54). It is the most effective treatment option for dyssynergic defecation, with studies showing 70%–80% efficacy and symptom remission lasting over 2 years (76). In patients with functional defecation disorders and quiescent IBD, studies suggest that biofeedback therapy is effective in 70% of patients (11). Additionally, it can improve functional defecation disorder symptoms in patients with ileo-anal pouch anastomosis (11).

**Figure 2:**
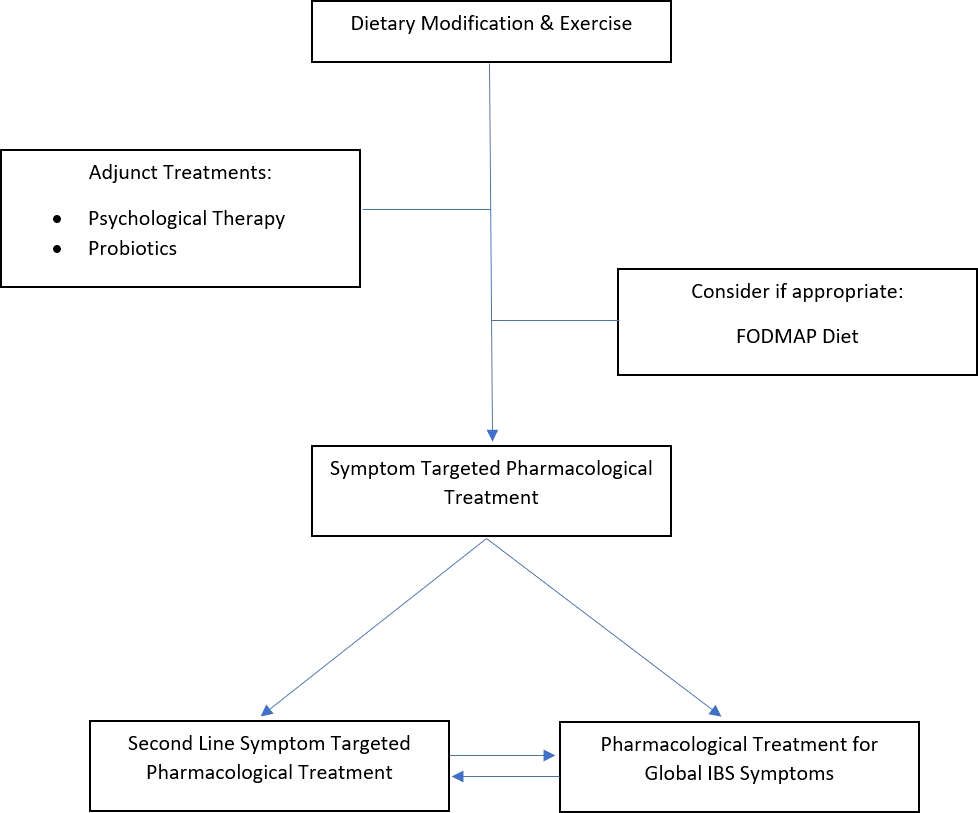
Suggested treatment algorithm for IBS symptoms in IBD.

In summary, concurrent FGIDs with IBD are best managed with a combination of dietary, lifestyle, psychological, and pharmacological therapies, that are tailored for safety in a patient whose gastrointestinal tract has undergone structural and functional changes caused by IBD (**Figure 2**).

## Discussion and conclusion

There is increasing recognition of the role that FGIDs play in causing refractory symptoms and detrimentally affecting quality of life in IBD. However, their identification and diagnosis are often delayed, and their management is based on empirical FGID treatments. Further research into the pathogenesis, specific diagnostic markers, and tailored therapies for FGIDs in IBD is needed.

## Author contributions

CW, AB, AC, and AO’C contributed to conception and design of the review. CW wrote the first draft of the manuscript. CW, AB, and AO’C wrote sections of the manuscript. All authors contributed to manuscript revision, read, and approved the submitted version.

## Conflict of interest

The authors declare that the research was conducted in the absence of any commercial or financial relationships that could be construed as a potential conflict of interest.

## Publisher’s note

All claims expressed in this article are solely those of the authors and do not necessarily represent those of their affiliated organizations, or those of the publisher, the editors and the reviewers. Any product that may be evaluated in this article, or claim that may be made by its manufacturer, is not guaranteed or endorsed by the publisher.

## Abbreviations

ACTH, adrenocorticotrophin; Anti-TNFα, anti-tumor necrosis factor α; BAD, bile acid diarrhea; CBT, cognitive behavioral therapy; CD, Crohn’s disease; CRP, C-reactive protein; FGID, functional gastrointestinal disorder; FODMAP, fermentable oligo-, mono-, and disaccharides and polyols; GABA, ƴ-aminobutyric acid; IBD, inflammatory bowel disease; IBS, irritable bowel syndrome; IBS-D, irritable bowel syndrome predominant diarrhea; NICE, National Institute for Clinical Excellence; NSAID, nonsteroidal anti-inflammatory drug; SIBO, small intestinal bacterial overgrowth; SNRI, serotonin-norepinephrine reuptake inhibitor; SSRI, selective serotonin reuptake inhibitor; TCA, tricyclic antidepressant; UC, ulcerative colitis.
